# Circadian regulation of developmental synaptogenesis via the hypocretinergic system

**DOI:** 10.1038/s41467-023-38973-w

**Published:** 2023-06-02

**Authors:** Xu-Fei Du, Fu-Ning Li, Xiao-Lan Peng, Bing Xu, Yu Zhang, Guang Li, Taole Liu, Ying Li, Han Wang, Jun Yan, Jiu-Lin Du

**Affiliations:** 1 Institute of Neuroscience, State Key Laboratory of Neuroscience, Center for Excellence in Brain Science and Intelligence Technology, Chinese Academy of Sciences, 320 Yue-Yang Road, 200031 Shanghai, China; 2 University of Chinese Academy of Sciences, 19A Yu-Quan Road, 100049 Beijing, China; 3 Center for Circadian Clocks, Soochow University, 215123 Suzhou, Jiangsu China; 4 School of Biology and Basic Medical Sciences, Suzhou Medical College, Soochow University, 215123 Suzhou, Jiangsu China; 5 School of Life Science and Technology, ShanghaiTech University, 319 Yue-Yang Road, 200031 Shanghai, China

**Keywords:** Synaptic development, Circadian regulation

## Abstract

The circadian clock orchestrates a wide variety of physiological and behavioral processes, enabling animals to adapt to daily environmental changes, particularly the day-night cycle. However, the circadian clock’s role in the developmental processes remains unclear. Here, we employ the in vivo long-term time-lapse imaging of retinotectal synapses in the optic tectum of larval zebrafish and reveal that synaptogenesis, a fundamental developmental process for neural circuit formation, exhibits circadian rhythm. This rhythmicity arises primarily from the synapse formation rather than elimination and requires the hypocretinergic neural system. Disruption of this synaptogenic rhythm, by impairing either the circadian clock or the hypocretinergic system, affects the arrangement of the retinotectal synapses on axon arbors and the refinement of the postsynaptic tectal neuron’s receptive field. Thus, our findings demonstrate that the developmental synaptogenesis is under hypocretin-dependent circadian regulation, suggesting an important role of the circadian clock in neural development.

## Abstract (web summary)

Whether the circadian clock regulates early developmental processes is poorly understood. Here, the authors report the circadian rhythm of synapse formation during early brain development by using the retinotectal system of larval zebrafish as an in vivo model.

## Introduction

The circadian rhythm is a general timing property of biological processes^1^. Under physiological conditions, the circadian clock coordinates the functions of neural, endocrine, cardiovascular, immune, metabolic as well as other biological systems^2–5^. Although the circadian clock is established during early development^6,7^, its roles in regulating developmental processes are largely unexplored^8,9^, except that the circadian clock was found to affect the timing of cell cycle in larval zebrafish^10,11^, eclosion in *Drosophila*^12^ and kidney organogenesis in mammals^13^. During neural development, the synaptogenesis is an indispensable process for establishing neural circuits and can be regulated by multiple non-cell-autonomous factors including environmental cues^14,15^.

In the present study, using the larval zebrafish as an in vivo animal model, we found that the development of retinotectal synapses, which are formed by retinal ganglion cell (RGC) axons on tectal neuron (TN) dendrites in the neuropil of the optic tectum (OT), is under circadian regulation. Mechanistically, hypocretinergic neurons in the lateral hypothalamus, of which the axon terminals undergo both circadian and homeostatic synaptic plasticity in more mature larval zebrafish^16^, is essential for this circadian synaptogenesis rhythmicity. Functionally, the circadian synaptogenesis rhythm is involved in the arrangement of synaptic puncta on presynaptic axon arbors and the refinement of postsynaptic neurons’ visual receptive fields. Together, our work demonstrates that the circadian clock can regulate synapse growth during neural development, indicating the general influence of circadian clock on developmental processes.

## Results

### Circadian rhythm of developmental synaptogenesis

**Fig. 1:**
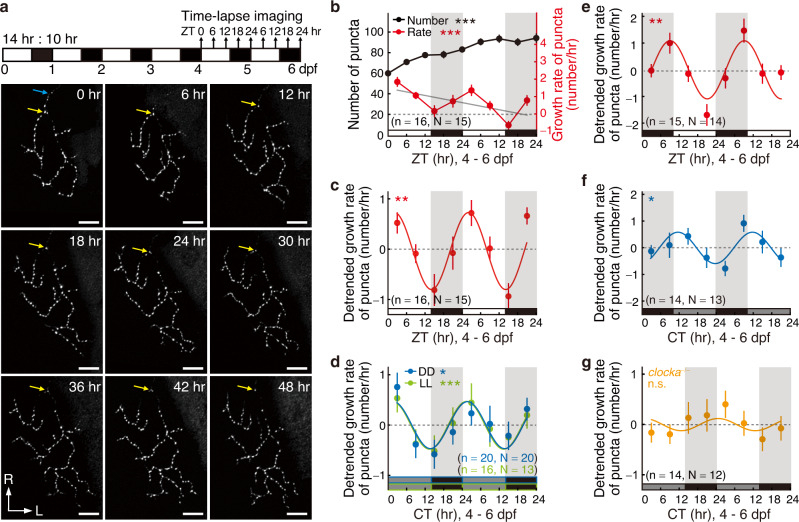
Circadian rhythm of retinotectal synaptogenesis during development in larval zebrafish in vivo. **a** Top, experimental procedure diagram. *PGUSG* larvae were entrained to 14–10 light-dark (LD) cycles during 0–4 days post fertilization (dpf) and then imaged every 6 hrs for 2 days from light onset (ZT0) at 4 dpf. Bottom, images obtained with a 6 h interval of a typical RGC axon arbor expressing Sypb-EGFP in the right hemisphere of the optic tectum (OT). Blue arrow, the primary axon; yellow arrow, a punctum at the first branch point of the axon arbor. L, lateral; R, rostral. Scale bars: 10 µm. **b** Summary of the total number (black) and growth rate (red) of Sypb-EGFP puncta on single-RGC axon arbor during 4–6 dpf. The larvae were under LD during 0–6 dpf. Gray line, linear-fitting line on the mean value of growth rate data. **c** Detrended growth rate of puncta shown in (**b**, red). It was obtained by subtracting the linear-fitting line from each raw data. **d**–**g** Detrended growth rate of puncta imaged during 4–6 dpf for wild type (WT) (**d**–**f**) or *clocka*^−/−^ (**g**) larvae raised under different light conditions. **d** Blue and (**g**), LD during 0–4 dpf and constant darkness (DD) during 4–6 dpf; **d** Green, LD during 0–4 dpf and constant light (LL) during 4–6 dpf; **e** dark-light (DL) during 0–6 dpf; **f** DL during 0–4 dpf and DD during 4–6 dpf. Zebra stripes, day-night cycles; white stripe, daytime; gray stripe, subjective daytime; black stripe, nighttime or subjective nighttime. Colored curve, cosine-fitting waves. CT, circadian time; ZT, zeitgeber time. Numbers in brackets indicate the number of RGCs (n) and larvae (N) examined. n.s., not significant; ***P < 0.0001 in (**b**), **P = 0.001 in (**c**), *P = 0.01 and ***P = 0.0006 in (**d**), **P = 0.006 in (**e**), *P = 0.01 in (**f**) (one-way ANOVA for (**b**), one-tailed Fisher’s *g*-test for (**c**–**g**)). Error bars denote s.e.m.. Source data are provided as a Source Data file.

To monitor developmental synaptogenesis over prolonged periods in the brain of living animals, we performed in vivo long-term time-lapse two-photon imaging of the double transgenic larval zebrafish *Tg(pou4f3:GAL4-VP16)ion6d;Tg(UAS:sypb-EGFP)ion7d (PGUSG)*^17^, in which the presynaptic marker synaptophysin fused with enhanced green fluorescent protein (Sypb-EGFP) is expressed on the axon arbor of *pou4f3* + (also known as *brn3c*) retinal ganglion cells (RGCs). The Sypb-EGFP has been successfully used to monitor the developmental dynamics of the presynaptic puncta of retinotectal synapses in zebrafish^18^. We previously also validated the transgenically-expressed Sypb-EGFP as a presynaptic marker by showing that about 88% of Sypb-EGFP-positive puncta were co-localized with the presynaptic marker SV2^17^. Due to the position-effect variegation of GAL4/UAS-transgene^19^, the *PGUSG* presents mosaic labeling of RGCs by Sypb-EGFP^17^. Therefore, we can trace all the presynaptic puncta on the axon arbor of individual RGCs in the transgenic fish siblings with sparse labeling of single or very few RGCs’ axon arbors in one hemisphere of the OT (Supplementary Fig. 1a). Through daily imaging of the axon arbor of individual RGCs expressing Sypb-EGFP, we found that the total number of presynaptic puncta increased rapidly during 3–6 days post fertilization (dpf) and gradually reached a plateau during 7–9 dpf (Supplementary Fig. 1b, c). Interestingly, imaging at a 6 h interval over the rapid growth period of 4–6 dpf showed that the increase in the number of synapses was higher during daytime than that during nighttime (Fig. 1a and red line in Fig. 1b). This day-night variation of synapse growth rate was superimposed upon a day-to-day increase in the total number of synapses (Fig. 1b, black line). Moreover, this daily oscillation in synapse growth rate was maintained with a dampened amplitude through the whole developmental period examined (4–6 dpf in Fig. 1b red line, 7–9 dpf in Supplementary Fig. 2). During development, there is a decreasing trend of the rate of synapse growth (Fig. 1b gray line and Supplementary Fig. 3). After removing the trend to make the fluctuation component prominent, the oscillating growth rate was transformed into an oscillating detrended growth rate (punctum number change per hour relative to the baseline trend), which could be well fitted by a sinusoidal function with a ~ 24 h cycle (Fig. 1c and Supplementary Table 1, *P* < 0.01). We thus used this detrended growth rate for all the following rhythmicity analysis. These results indicate that the retinotectal synapse growth undergoes diurnal fluctuation.

To test whether this diurnal oscillation of synaptogenic rate is controlled by the circadian clock, we monitored the developmental process of retinotectal synapses in larvae that were kept under constant darkness (DD) or constant light (LL) during 4–6 dpf to remove the entrainment effect of environmental illumination changes. The larvae were kept under regular light-dark (LD) cycles before 4 dpf for establishing the normal circadian rhythm. We found that the rhythmic oscillation of synaptogenic rate was preserved under both DD and LL conditions (Fig. 1d and Supplementary Table 1, *P* < 0.05 for DD, *P* < 0.001 for LL). Meanwhile, the synaptogenic rate rhythmicity was entrained in larvae which were kept in the reversed LD cycles (i.e., dark-light, DL conditions) during 0–6 dpf (Fig. 1e and Supplementary Table 1, *P* < 0.01), and the reset rhythm was also preserved under DD conditions during 4–6 dpf (Fig. 1f and Supplementary Table 1, *P* < 0.05).

Furthermore, we tested whether the synaptogenic rate rhythmicity is affected in the clock gene mutant *clocka*^−/−^ (also known as *clock1a*). To verify that the clock function is disrupted in the visual system of *clocka* mutants, we examined the expression of the clock gene *per1b* in the OT by whole-mount in situ hybridization (WISH) at two circadian time points relating to its peak and trough expression levels^20^. We found that the *per1b*’s expression level and the amplitude of its circadian expression rhythm both were largely reduced in the *clocka* mutant (Supplementary Fig. 4), indicating the perturbation of clock function. In the *clocka* mutant, the circadian rhythm of synaptogenic rate was disrupted (Fig. 1g and Supplementary Table 1, *P* = 0.3). Taken together, these results indicate the involvement of the circadian clock in regulating developmental retinotectal synaptogenesis.

**Fig. 2:**
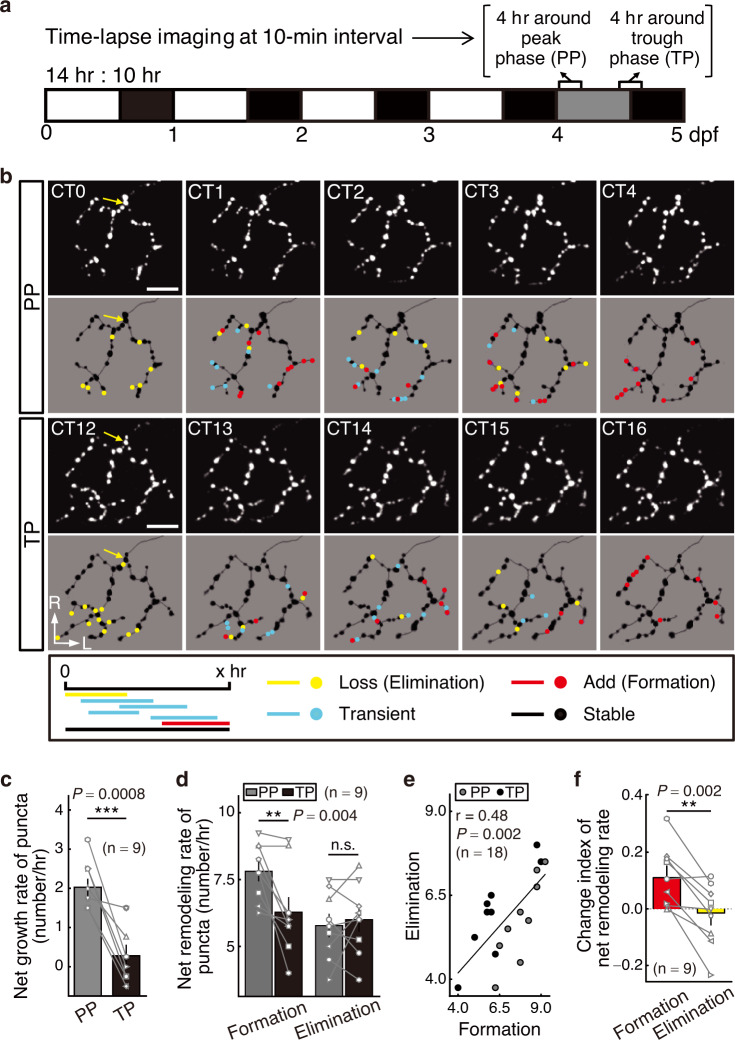
Circadian regulation of synapse formation but not elimination. **a** Experimental procedure diagram. *PGUSG* larvae entrained with LD cycles during 0–4 dpf were then transferred to DD for 1 day, and in vivo time-lapse two-photon imaging at a 10 min interval was performed from CT0 - CT4 and CT12 - CT16 on the same RGC axon arbor during 4–5 dpf. **b** Typical time-lapse images showing the growth dynamics of all Sypb-EGFP puncta on a single-RGC axon arbor within PP and TP during 4–5 dpf. In the lower panels, punctum images are superimposed on the reconstructed RGC axon arbor and the puncta are color-coded according to their fate defined as shown in the rectangle below and depicted in the Methods. Here, only time series at 0-, 1-, 2-, 3- and 4 h time points were shown. To real-timely display the punctum fate, for each time point, puncta were color-coded according to their fate determined by considering within the 2 h time window of 1 h before and after. Yellow arrow, the punctum at the first branch point of the RGC axon arbor. L, lateral; R, rostral. Scale bars, 10 μm. **c**, **d** The net growth rate (**c**) and the net remodeling (including formation and elimination) rate (**d**) of Sypb-EGFP puncta on individual RGC axon arbors over 4 h within PP (gray) and TP (black). **e** Scatterplot shows the correlation between the net formation and elimination rate during PP and TP. Black line, linear-fitting line on the 18 data points from 9 cells. **f** Change index ((Rate_PP_$-$Rate_TP_)/(Rate_PP_$+$Rate_TP_)) of net formation and elimination remodeling rate. Centre, median; bounds of box, first and third quartiles; whiskers, minimum and maximum values. The number in the brackets indicates the number of RGCs examined, and each RGC was imaged from an individual larva. PP peak phase, TP trough phase. n.s., not significant; **P < 0.01, ***P < 0.001 (two-tailed paired Student’s *t* test for (**c** and **f**), two-way ANOVA and Bonferroni’s multiple comparisons test for **d**). Error bars denote s.e.m.. Source data are provided as a Source Data file.

### Synapse formation rather than elimination exhibits circadian rhythmicity

The developmental synaptogenesis is a highly dynamic process consisting of concurrent events of synapse formation and elimination^21^. To examine which developmental event underlies the rhythmicity of synaptogenic rate, we performed the time-lapse imaging with a 10 min interval of individual RGC axon arbors expressing Sypb-EGFP in larvae aged between 4–5 dpf over two 4 h periods (CT0 - CT4 and CT12 - CT16; CT, circadian time) (Fig. 2a, b), which respectively span the growth peak phase (PP) during daytime (CT1.83) and trough phase (TP) during nighttime (CT13.83) (see Fig. 1d and Supplementary Table 1). The larvae examined were kept under regular LD cycles before 4 dpf for establishing normal circadian rhythm and then transferred to DD to remove the entrainment effect of environmental illumination changes.

The high temporal-resolution imaging allows us to track the fate of all the puncta that preexisted at the beginning of imaging and that newly formed during imaging (Fig. 2b and see Methods)^18,21^. We first analyzed the lifetime distribution of new Sypb-EGFP puncta and found a similar average punctum lifetime of 51.2 ± 1.9 min (*n* = 783, mean ± s.e.m.) and 52.2 ± 2.0 min (*n* = 662, mean ± s.e.m.) during PP and TP (*P* = 0.7), respectively (Supplementary Fig. 5). The lifetime of over one-half of the puncta was <30 min, indicating a high synapse dynamics during development. Then we quantified the net growth rate of puncta over the total 4 h time window, and consistently found a higher rate in PP than in TP (Fig. 2c, *P* < 0.001; see also Fig. 1b, red line). Importantly, this higher growth rate during PP was mainly contributed by more punctum formation rather than less punctum elimination (Fig. 2d, *P* < 0.01). In addition, for individual cells, the net formation and elimination rate were positively correlated (Fig. 2e, *P* < 0.01), indicating coordinate dynamics of synapse formation and elimination. Further analysis revealed that the net rate change of punctum formation between PP and TP, calculated as change index: (Rate_PP_$-$Rate_TP_)/(Rate_PP_$+$Rate_TP_), was significantly larger than that of punctum elimination (Fig. 2f, *P* < 0.01), of which the index was around zero. Taken together, these results suggest that synapse formation is under circadian regulation.

### Requirement of the hypocretinergic signaling

**Fig. 3:**
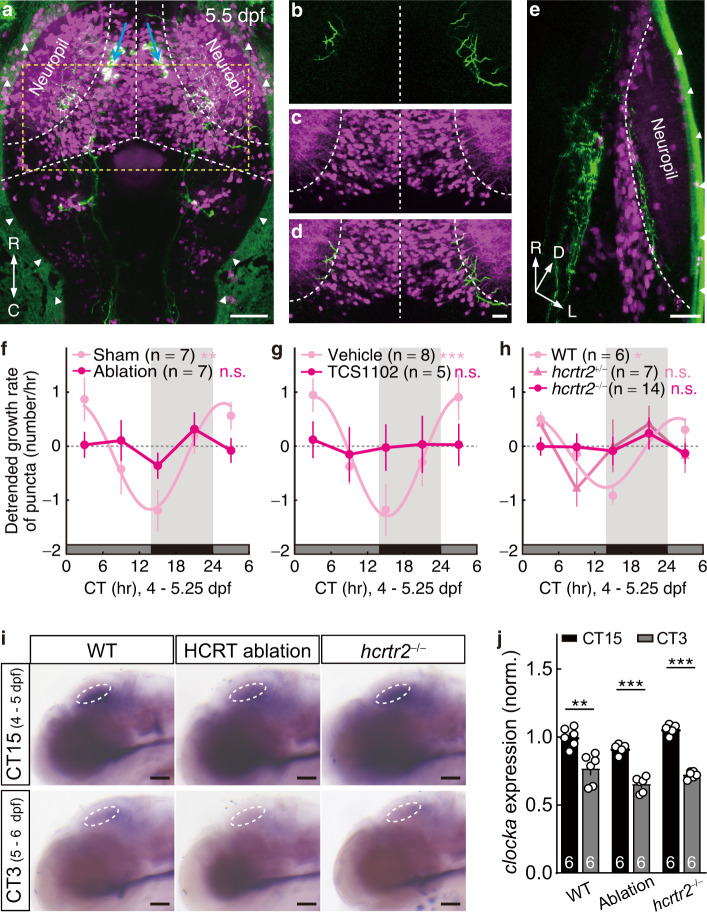
Hypocretinergic system is required for the circadian rhythm of synaptogenic rate. **a**–**e** Confocal images of the dorsal projection view (**a**), a cropped and partial (6-μm thick) projection view (**b**–**d**), and the rotated (60° along *y*-axis) lateral 3-D projection view (**e**) of a 5.5-dpf *Tg(−2.0Tru.Hcrt:EGFP)zf11;Mü4023* larva showing the location of HCRT neuron somas (**a**, blue arrows) and their axon projections (green, **b**–**e**). The *Mü4023* labels some tectal neurons (TNs) in OT (magenta). Arrowheads, nonspecific fluorescent signals on the skin; white dotted line, borders of the OT and the boundaries between the neuropil and soma layer of the OT or between the two tectal hemispheres; yellow dotted line, the cropped area for (**b**–**d**). Three independent experiments were repeated. **f**–**h** Detrended growth rate of Sypb-EGFP puncta imaged during 4–5.25 dpf showing that the circadian rhythm of retinotectal synaptogenesis is disrupted by bilateral HCRT neuron ablation (**f**), HcrtR antagonist TCS1102 treatment (**g**), and *hcrtr2* mutation (**h**). Larvae entrained with normal LD cycles during 0–4 dpf were then reared under DD. **i** Lateral view of whole mount in situ hybridization showing unaffected rhythmic *clocka* expression under DD conditions after 4-day LD entrainment in the OT of larvae with bilateral HCRT neuron ablation or *hcrtr2* mutation. **j** Quantification of the *clocka* mRNA expression in OT at the two circadian time points shown in (**i**). For each individual, the mean signal intensity in the circled tectal area (white circle in **i**) was normalized to the mean value of the WT group at CT15. The numbers on the bars indicate the numbers of larvae examined. Scale bars, 50 μm (**a**, **i**), 20 μm (**b**–**e**). n.s., not significant; **P = 0.002 in (**f**), ***P = 8.0 × 10^−4^ in (**g**), *P = 0.01 in (**h**); **P = 0.001, ***P_Ablation_ = 2.0 × 10^−6^ and ***P_*hcrtr2*_^-/-^ < 1.0 × 10^−6^ in (**j**) (one-tailed Fisher’s *g*-test for (**f**–**h**), nonparametric two-tailed multi *t*-test wi*t*h Holm-Sidak method for multiple comparisons correction for **j**). C caudal, D dorsal, L lateral, M medial, R rostral, CT circadian time. Error bars denotes s.e.m.. Source data are provided as a Source Data file.

We next examined how the circadian regulation of synaptogenesis occurs. We interrogated hypocretin (HCRT, also known as orexin), a sleep/wake regulator released by the hypocretinergic neurons in the lateral hypothalamus^22^. In larval zebrafish, the axon terminals of HCRT neurons were reported to undergo circadian structural plasticity^16^, indicating the rhythmic release of Hcrt peptides in the brain. Confocal imaging of the double transgenic larvae *Tg(−2.0Tru.Hcrt:EGFP)zf11;*^23^*Mü4023*^24^, in which HCRT neurons and some TNs express EGFP and mCherry, respectively, revealed bilateral hypocretinergic axon projections to the stratum album centrale/stratum periventricular (SAC/SPV) retinorecipient layer of the OT (Fig. 3a–e), which locates ventral to the retinorecipient layer of *pou4f3* + RGCs’ main projections at the stratum fibrosum et griseum superficiale (SFGS)^25^.

To explore the requirement of the HCRT neural system for the rhythmic synaptogenesis, we disrupted the HCRT signaling via three ways. First, we depleted all HCRT neurons by two-photon laser-based ablation at 3.5 dpf (Supplementary Fig. 6). Second, we blocked the hypocretin receptor 2 (HcrtR2) with HcrtR antagonist TCS1102 (30 μM)^26,27^. Third, we generated a HcrtR2 null mutant *hcrtr2*^*ion28d*^ (Supplementary Fig. 7). We found that, without significant changes in the growth rate of Sypb-EGFP puncta at the end of the observation time (Supplementary Fig. 8), all three manipulations abolished the circadian rhythm of synaptogenic rate (Fig. 3f–h and Supplementary Table 1). To test whether HCRT signal directly or indirectly acts on those *pou4f3* + RGCs examined, we performed the WISH of *hcrtr2* and found that *hcrtr2* was indeed expressed in the RGC layer (Supplementary Fig. 9a, b). However, by using whole-mount fluorescent in situ hybridization (FISH) of *hcrtr2* in combination with anti-GFP immunostaining on *Tg(pou4f3:GAL4-VP16);Tg(4×nrUAS:GFP)* fish, we further found that the *pou4f3* and *hcrtr2* largely expressed in different cells in the ganglion cell layer (Supplementary Fig. 9c). These results suggest an indirect but essential function of HCRT signaling in the circadian rhythm of retinotectal synaptogenesis.

To examine whether the HCRT signaling functions upstream or downstream of the clock mechanism, we quantified the rhythmic expression of *clocka* by WISH in the OT and the RGC layer in the larvae with HCRT neuron ablation or *hcrtr2* mutation. We found that, in accordance with a previous report^28^, the endogenous *clocka* expression was high in the early night at CT15 and low near dawn at CT3 in normal larvae. Notably, this rhythmicity was not impaired by either of the two manipulations (Fig. 3i, j and Supplementary Fig. 9d, e), implying the HCRT signaling operates downstream of the clock mechanism to regulate circadian synaptogenesis rhythm.

### Implications of circadian synaptogenesis rhythm

**Fig. 4:**
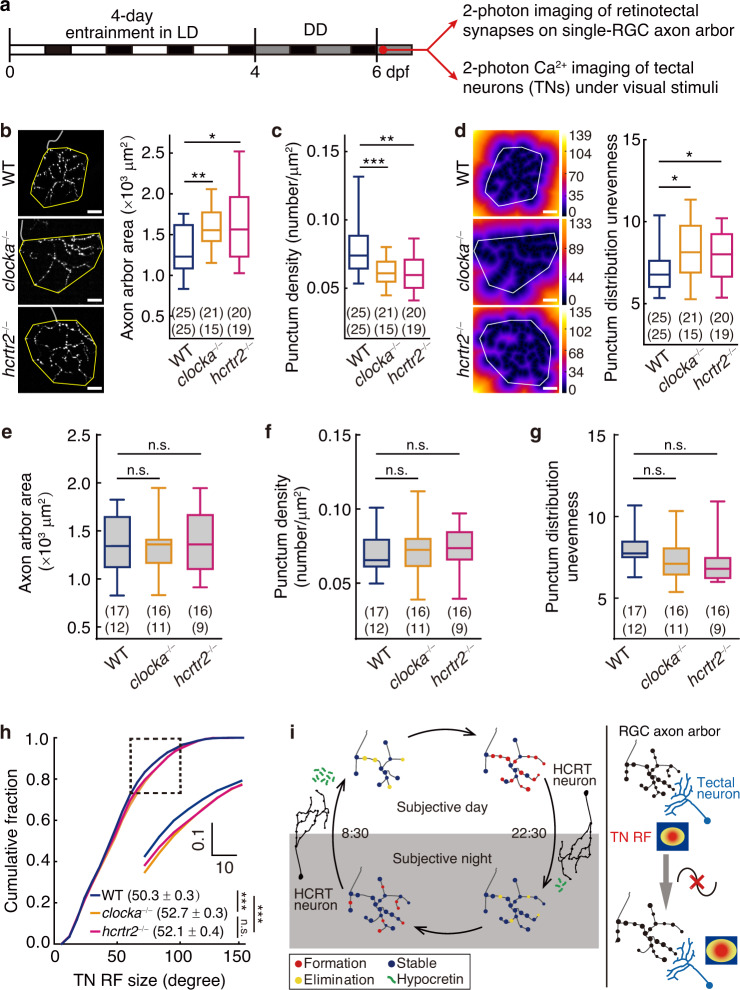
Circadian rhythm of synaptogenesis affects the arrangement of retinotectal synapses and the refinement of visual function. **a** Experimental procedure diagram. Larvae reared under LD during 0–4 dpf and DD afterwards were assayed at 6 dpf. **b** Left, projection view of a typical RGC axon arbor expressing Sypb-EGFP in WT, *clocka*^−/−^ or *hcrtr2*^−/−^. Right, boxplots of the arbor territory/area (yellow polygon) (**P = 0.006; *P = 0.02). **c** Boxplots of the punctum density of single-RGC axon arbor calculated by total punctum number dividing arbor territory (***P = 0.0006; **P = 0.001). **d** Left, heat maps of the images in (**b**) color-coded by the distance of each pixel to its nearest punctum. Right, boxplots of the standard deviation of pixel intensities within the arbor territory (white polygon) (*P* = 0.02). **e**–**g** The same measurements as in (**b**–**d**) but larvae were raised in DD from 0 dpf. **h** Cumulative distribution of the receptive field (RF) size of tectal neurons (TNs) in WT (5976 TNs, 12 larvae), *clocka*^−/−^ (5416 TNs, 12 larvae) and *hcrtr2*^−/−^ (4859 TNs, 10 larvae) (*P*_*clocka*_^-/-^_vs WT_ = 5.2 × 10^−8^; *P*_*hcrtr2*_^-/-^_vs WT_ = 1.2 × 10^−4^). Inset, zoom-in view of the dashed black square. **i** Working model. Left, during brain development, endogenous circadian clock drives retinotectal synapses (made by RGC axons on TN dendrites) to grow faster at daytime and slower at nighttime, a process due to rhythmic synapse formation (red puncta) but not synapse elimination (yellow puncta). The HCRT neural system is involved in this circadian retinotectal synaptogenesis rhythm. Right, disruption of the circadian synaptogenesis rhythm causes defects in the development of retinotectal synapses, RGC axon arbors and TN function. Scale bars, 10 μm. Numbers in brackets indicate the numbers of RGCs (top) and fish (bottom) examined. The same datasets were used in (**b**–**d**) or in (**e**–**g**). n.s., not significant; *P < 0.05, **P < 0.01, ***P < 0.001 (nonparametric two-tailed Mann–Whitney test in (**b**–**g**); one-tailed two-sample Kolmogorov-Smirnov test in (**h**)). Boxplots in (**b**–**g**): centre, median; bounds of box, first and third quartiles; whiskers, minimum and maximum values. Source data are provided as a Source Data file.

To examine the role of the circadian synaptogenesis rhythm in the development of the retinotectal system, we first analyzed the change in structural features of synapses and RGC axon arbors in both the *clocka*^−/−^ and *hcrtr2*^−/−^ after 2-day free-running in DD at 6 dpf and 4-day free-running in DD at 8 dpf (Fig. 4a). Previous study has shown that the length of RGC axon arbors labeled by GFP and Sypb-EGFP is comparable and growths in a very similar developmental time course, indicating concurrent axon arbor growth and synaptogenesis^18^. Therefore, based on the Sypb-EGFP punctum distribution, we reconstructed the axon arbor morphology and measured the total punctum number, axon arbor size (total arbor length and arbor area), and arbor complexity (branch number) (Left in Fig. 4b and Supplementary Fig. 10a). When measured at 6 dpf, the total punctum number, axon arbor length and branch number in both the mutants were not obviously changed in comparison with the wild type (WT) siblings (Supplementary Fig. 10b–d), but the axon arbor area was significantly increased (Fig. 4b, *P* < 0.01 for WT vs *clocka*^−/−^, *P* < 0.05 for WT vs *hcrtr2*^−/−^), leading to a decreased punctum density (Fig. 4c, *P* < 0.001 for WT vs *clocka*^−/−^, *P* < 0.01 for WT vs *hcrtr2*^−/−^). However, the three unchanged parameters at 6 dpf were found to be significantly changed when examined at 8 dpf (Supplementary Fig. 10e–g), implying an accumulated effect of disrupted circadian clock on these structural features. Furthermore, the puncta distributed more unevenly within the axon arbor area in the two mutants (Fig. 4d, *P* < 0.05), suggesting that the arrhythmic development leads to inhomogeneous mesh sizes of the axon arbor^29^. In summary, the synapse arrangement on axon arbors and the arbor structure are both perturbed by the disruption of circadian synaptogenesis.

To exclude the possibility of circadian clock-independent regulation of *clocka* and *hcrtr2* on synaptogenesis and arbor growth, we raised the WT, *clocka*^−/−^ and *hcrtr2*^−/−^ larvae under DD conditions from fertilization (0 dpf). Previous studies have shown that raising in DD from fertilization can effectively abolish the establishment of circadian rhythms of clock gene expression and melatonin release in zebrafish^6,30^. We found that, under DD conditions from fertilization, both *clocka*^−/−^ and *hcrtr2*^−/−^ larvae showed no significant difference with the WT group (Fig. 4e–g), consistent with the notion that changes in retinotectal synaptogenesis in *clocka*^−/−^ and *hcrtr2*^−/−^ larvae are mainly circadian rhythmicity-dependent.

We next investigated the functional significance of the circadian synaptogenesis rhythm. At 6 dpf under DD conditions after 4-day LD entrainment, we mapped the receptive field (RF) of postsynaptic TNs^31^ by using in vivo Ca^2+^ imaging in the presence of monocular visual stimulation (Supplementary Fig. 11), in the larvae of *clocka*^−/−^ or *hcrtr2*^−/−^ with the transgene background of *Tg(elavl3:GCaMP6s)jf4*^32^. Consistent with the expanded axon arbor area of RGCs (see Fig. 4b), the RF size of TNs in both the mutants was significantly larger than that in WT larvae (Fig. 4h, *P* < 0.001).

Taken together, these results suggest that the circadian rhythm of synaptogenesis is important for the synapse arrangement development and visual function refinement in the retinotectal system.

## Discussion

During development, the animal body is constructed through temporally coordinated division, differentiation, proliferation, movement and interaction of cells. As an endogenous timing system, whether and how circadian information is implemented during development is crucial for understanding tissue assembly and organogenesis. In this study, we discovered the HCRT system-dependent circadian regulation of developmental synaptogenesis, which is critical for the structural arrangement and the functional development of neural circuits (Fig. 4i). This work extends the functional spectrum of the circadian clock to developmental processes and provides a layer of regulation that may coordinate developmental timing of multiple neural circuitries.

We mechanistically examined the involvement of the sleep/wake regulator HCRT in the circadian synaptogenesis rhythm, because previous studies showed that the activities of these neurons changed with active-resting cycles in mammals and fish^33,34^, and the axon terminals of HCRT neurons underwent circadian structural plasticity in zebrafish^16^. We revealed that Hcrt-HcrtR signaling plays an essential role in establishing the circadian synaptogenic rate rhythm of retinotectal synapses. Notably, the circadian synaptogenic rate rhythm was not only completely disrupted in the *hcrtr2* homozygous mutant (see Fig. 3h, *P* = 0.2 for *hcrtr2*^−/−^) but also partially blocked in the heterozygous mutant (see Fig. 3h, *P* = 0.06 for *hcrtr2*^+/−^), indicating a dose-dependent regulation by Hcrt. In addition, for the *pou4f3* + RGCs we examined, the lack of both the HcrtR expression and direct HCRT neuronal innervation makes it unlikely that the Hcrt-HcrtR signaling functions through direct peptide-receptor interactions on RGCs. Given the vicinity of HCRT neuronal innervation to TNs in the OT and the wealth brain targets of Hcrt^23^ (see Supplementary Fig. 9a), the signal might function through indirect retrograde feedback from TNs and/or indirect systemic pathways involving other brain nuclei. Whether the synaptogenic rate rhythm also exists in *pou4f3−* RGCs and other neurons in the brain, and whether the HCRT signaling is involved directly or indirectly are worthy of further examination. The requirement of a sleep/wake regulator in the maintenance of circadian rhythms provides a link for the interaction and crosstalk between the circadian and sleep/wake (brain-state) regulation^35^. And the HCRT system may act as a clock output pathway that relay circadian temporal information from the circadian clock to other downstream effectors.

It is interesting that the rising phase of the synaptogenic rate rhythm is during the late subjective night (Fig. 1c, d). We speculate that the synapse formation event dominates during the beginning period of the day to provide a large immature synapse pool, in which the correct synapse will be stabilized and the incorrect ones will be eliminated during the daytime, and the preparation for this pool may start earlier from the subjective night. As there are more visual experiences during the daytime, this speculation is consistent with the notion of the visual experience-dependent development of the visual system.

In accordance with that the enhanced visual activity driven by light promotes dendrite and synapse growth^36,37^, the oscillation amplitude under normal LD/DL conditions (0.9 on average) is larger than that under DD/LL conditions (0.7 on average), indicating that light may also be a driving force for the oscillation under ambient lighting conditions. Considering that the establishment of neural circuits is a ‘trial-and-error’ process^21^, we propose that the selective regulation by the circadian clock on synapse formation at daytime is an efficient strategy for activity-dependent circuit wiring, because nascent synapses tend to be correct and stabilized at daytime with enriched visual experience, and thus undergo less frequently energy-consuming ‘trial-and-error’ processes. Therefore, the disruption of this temporal coordination results in defects in both the synapse arrangement on axon arbors and neuronal function development, revealing the significance of the matched timing of the circadian synapse formation with the light-dependent visual experience in the visual system. Based on the synaptotrophic hypothesis that synapses promote new branch formation and stabilize correct new branches^18,38^, circadian synapse formation may instruct the axon arbors of RGCs as well as the dendrites of TNs to undergo a likewise circadian elaboration during development.

## Methods

### Animals

Adult zebrafish (*Danio rerio*) were maintained in an automatic fish housing system (ESEN, Beijing, China) at 28 °C under a 14 h light: 10 h dark cycle. The larval zebrafish used were in *nacre* background and reared with 10% Hank’s solution. The transgenic lines used were listed below (abbreviated names are in parentheses): *Tg(pou4f3:GAL4-VP16)ion6d;Tg(UAS:sypb-EGFP)ion7d* (*PGUSG*)^17^, *PGUSG;clocka*^−/−^, *Tg(−2.0Tru.Hcrt:EGFP)zf11*^23^*;Mü4023*^24^, *Tg(−2.0Tru.Hcrt:EGFP)zf11*^23^*;PGUSG*, *PGUSG;hcrtr2*^*ion28d*^ (made in the present study), *Tg(pou4f3:GAL4-VP16)ion6d;Tg(4×nrUAS:GFP)c369*^39^ (*PGUG*), *Tg(elavl3:GCaMP6s)jf4*^31^*;clocka*^−/−^ and *Tg(elavl3:GCaMP6s)jf4*^31^*;hcrtr2*^*ion28d*^. All experimental procedures using live animals were performed in accordance with guidelines approved by the Animal Care and Use Committee of the Center for Excellence in Brain Science and Intelligence Technology, Chinese Academy of Sciences (NA-046-2023).

### Generation of mutant zebrafish

The type II bacterial clustered regularly interspaced short palindromic repeats (CRISPR)/CRISPR-associated (Cas) 9 (CRISPR/Cas9) system^40^ was used to generate the *hcrtr2* mutant (*hcrtr2*^*ion28d*^).

For generating the *hcrtr2* mutant, we designed a short guide RNA (sgRNA) targeting the first exon of the zebrafish *hcrtr2* gene (GenBank: NM_001079868.1) and co-injected the sgRNA (100 pg) with Cas9 (600 pg) protein into one-cell-stage embryos, which yields a cleavage efficiency of ~62%. Three mutations were generated for *hcrtr2*, and we used the mutation with 1 base-pair deletion, which leads to open reading frame change and a premature stop codon and thus a truncated protein (Supplementary Fig. 7). For genotyping, we collected genomic template DNA from the whole body of individual larvae or the tail fin of adults and designed allele-specific PCR followed by restriction-enzyme digestion to distinguish wild type (WT) and mutant alleles. The primer pairs used for genotyping are 5′ -CAGTATCGGAGATGCGCGATG-3′ and 5′ -CGCTGATGAGATGGTTTTGTGAGTT-3′ .

### In vivo time-lapse two-photon imaging of RGC axon puncta

For performing in vivo time-lapse two-photon imaging, *PGUSG* larvae with sparse labeling of RGC axon arbors on either side of the OT were prescreened during late daytime at 3 dpf and raised in a 24-well plate with individual larva in one well. The imaging was performed under a 40× water-immersion objective (N.A., 0.8) on an Olympus Fluoview 1000 microscope (Tokyo, Japan) with a two-photon laser (Newport/Spectra-Physics, USA) tuned to 900 nm for excitation of EGFP. This near-infrared excitation wavelength is essential for this study, because it is invisible to fish and avoids laser-induced changes in their circadian rhythm. During imaging, a heating system (CU-201, Live Cell Instrument) was used to keep the temperature of imaging chamber at ~28 °C. For daily or 6 h interval time-lapse imaging, larvae were anesthetized with 0.02% MS-222. For high temporal-resolution time-lapse imaging at a 10 min interval, larvae were immobilized via brief (~2 min) exposure to reversible paralytic pancuronium dibromide (PCD, 1 mM, Tocris 0693). Then the larvae were mounted in 1.5% low-melting agarose for imaging and were released immediately after imaging and reared under given light conditions. During each experiment, 15–20 larvae were imaged sequentially and the whole imaging was finished within 2 h around each sampling time point. For DD condition, all the operations were performed under dim red light, and the axon arbor of RGCs to be imaged was located by rapid two-photon laser-scanning in one tectal hemisphere with low laser intensity.

### Image analysis

The analysis of presynaptic puncta on RGC axon arbors was performed on the maximum-intensity projection of *z*-stack images. The projected images were median (3 × 3) filtered to reduce photomultiplier tube noise. Puncta were identified as discrete local accumulations of Sypb-EGFP signal of more than 2 native pixels (0.4 μm) in diameter and counted with Image-Pro Plus (Media Cybernetics) Count/Size function. Based on the punctum number distribution at 4 dpf (61.8 ± 13.2, mean ± s.d., *n* = 72) and in order to compensate developmental discrepancy of individual RGCs from individual larvae, the axon arbor on which the initial total number of puncta at 4 dpf falls in between 45 and 75 was chosen for analysis. Puncta were counted blindly (the experimenter was blinded to zeitgeber/circadian time, the phenotype of fish, or treatments). In Fig. 1a, z-stacks were de-convoluted using AutoQuant X software (Media Cybernetics) to create clearer images shown in the panels.

For analyzing punctum remodeling (formation and elimination), time-series projections with high temporal resolution (10 min interval) were merged into a new stack image and aligned with Fiji (Image J) (http://fiji.sc) MultiStackReg plugin. Then each Sypb-EGFP punctum was identified as aforementioned and manually tracked through time series to determine the punctum fate with Image-Pro Plus software. We defined the fate of puncta as four classes (see Fig. 2b, bottom rectangle): stable, existed throughout the imaging time window; elimination, preexisted but disappeared during imaging; formation, newly formed during imaging and maintained to the end; transient, newly formed during imaging but disappeared before the end of imaging. Therefore, the net growth of puncta (the total number at the end of 4 h imaging over that at the start) is the result of net punctum formation (the number of puncta that newly formed during imaging and maintained to the end) over net punctum elimination (the number of puncta that existed at the start of imaging but disappeared during imaging). With the presence of transient puncta, the total punctum remodeling level (punctum add and loss events) are far higher than the net punctum remodeling (net punctum formation and net punctum elimination). The net rate is the corresponding net change of punctum number divided by the observation time.

To analyze the spatial structural change of puncta on RGC axon arbors in the OT neuropil, the original image stack of Sypb-EGFP-expressing RGC axons was rotated in 3D and resliced into a new image stack in which all puncta are distributed in a plane, which was then projected to 2D. For analyzing the territory of punctum distribution on single-RGC axon arbors, we drew a polygon around the labeled Sypb-EGFP punctum field from the punctum at the first branch point and measured its area using Fiji. For analyzing the punctum distribution unevenness within the territory, we used the Interactive Geodesic Distance MAP plugin in Fiji, by which the intensity projection image was transformed into a heat map with each pixel encoding the distance of itself to the nearest Sypb-EGFP punctum location, and thus the coding shows a center-low-outer-high pattern globally. The distribution unevenness was calculated as the standard deviation of the distance value of all the pixels within the punctum-based RGC axon arbor territory.

To analyze the size and complexity of RGC axon arbors, based on Sypb-EGFP punctum signal, the skeletons of individual arbors were reconstructed semi-automatically by using the simple neurite tracer (SNT) plugin in Fiji. and saved as individual SWC files. Then the arbor length (representing arbor size) and branch number (representing arbor complexity) were measured by using the L-Measure software tool^41^.

### Confocal imaging

Confocal imaging was carried out under a 40× (N.A., 0.8) or 20× (N.A., 0.95) water-immersion objective with *z*-steps at 1.3–2 μm with an Olympus Fluoview 1000 or 3000 microscope (Tokyo, Japan). The green and red fluorophores were excited with 488 nm and 559 nm, respectively, under the sequential line-scanning mode.

### Whole-mount in situ hybridization of zebrafish embryos

The DNA fragments for synthesizing *per1b*, *hcrtr2* and *clocka* riboprobes were PCR amplified from fish cDNA with primers as follows (in 5′–3′): *per1b* F, TCTCGTGATTATCCAGCAGC; *per1b* R, AGCAGGTCCAGCAGATCACT; *hcrtr2* F, ATGTCCGGGATCTCCGTC; *hcrtr2* R, CTACACCGCCTGGTCTGAAAT; *clocka* F, AGTCTCAGTTGAACACCTCCAGC; *clocka* R, GCAGTGTGTGGGTCGACCTC. The digoxigenin (DIG) labeled antisense riboprobes were then transcribed in vitro using standard reagents (Roche 11277073910). Whole-mount in situ hybridization (WISH) was performed following a standard protocol^42^. Briefly, embryos at 2–5 dpf were treated with proteinase K (3 μg/ml, Roche 3115828001) for 20 min−1.5 h and hybridized with 1 ng/μl riboprobe overnight at 68–70 °C. Signals of in situ hybridization were developed by colorimetric staining (BCIP/NBT, Roche 11681451001). Stained embryos were mounted in glycerol and imaged using a microscope (LeicaM205FA) with a digital camera (LeicaDFC500), from which the resulting images were analyzed using Fiji. The fluorescent in situ hybridization (FISH) was performed using DIG-labeled riboprobes and the TSA System (Akoya Biosciences NEL744001KT) and operated before the anti-GFP immunofluorescence (primary antibody used was rabbit anti-GFP, 1:500, Thermo Scientific A-11122; secondary antibody used was goat anti-rabbit Alexa Fluor 488, 1:500, Molecular Probes A11034).

### Two-photon laser-based ablation of HCRT neurons

In *Tg(−2.0Tru.Hcrt:EGFP)zf11;PGUSG*, a two-photon laser (820 nm) was used to ablate EGFP-positive somas of HCRT neurons one by one in each hemisphere. The ablation was achieved by free-running line-scanning within the central optical section of the cell body. Successful ablation was determined when the target cell body showed a bulb-like structure in the bright-field image and lost EGFP fluorescence signal. The ablation was carried out on daytime around 3.5 dpf. At the end of time-lapse imaging experiments, the ablation effectiveness was checked and only the larvae with complete ablation of both HCRT neurons’ somas in the rostral hypothalamus and axon fibers in the OT were used for image analysis.

### Pharmacology

The HcrtR antagonist TCS1102 (Tocris 3818) was applied with a stock solution of 100 mM in DMSO diluted further in Hank’s solution to a working concentration of 30 μM. The *PGUSG* siblings with sparse RGC labeling were separated into two groups in 24-well plates and raised in the presence or absence of TCS1102 from CT0 of 4 dpf to the end of time-lapse imaging. The treatment solution was refreshed once every 24 h to compensate for potential degradation of the compound. The vehicle control larvae were treated with equivalent solvent only.

### Mapping the receptive fields of TNs

Larvae were entrained to LD cycles during 0–4 dpf, raised in DD for 2 days and then imaged at 6 dpf with two-photon fluorescence microscope. Larvae were paralyzed by α-bungarotoxin and embedded in 1.5% low-melting agarose on a customized chamber for monocular visual stimulation generated by a custom program written in MATLAB. Visual stimuli were presented onto the screen by a mini-projector covered with red filter paper to avoid spectrum interference^31^. The visual stimulation field was divided into 8 rows and 8 columns. The red moving squares (12° in size, 30°/s in speed) swept along each row and column with a 15 s interval. Each stimulus was randomly repeated twice.

The analysis was performed with a custom program written in MATLAB. Images were automatically aligned and segmented by Fiji. The averaged ΔF/F0 was calculated according to each stimulus. Responses at each intersection grid were calculated by multiplying the peak responses evoked by the vertical and horizontal stimuli sweeping through that location. The receptive field (RF) was fitted with a two-dimensional Gaussian model^43^:

1 $$f(x,y)={c}_{0}+{c}_{1}{e}^{(-\frac{{[(x-{c}_{2})\cos ({c}_{3})+(y-{c}_{4})\sin ({c}_{3})]}^{2}}{2{{c}_{5}}^{2}}\frac{{[-(x-{c}_{2})\sin ({c}_{3})+(y-{c}_{4})\cos ({c}_{3})]}^{2}}{2{{c}_{6}}^{2}})}$$

where $f\left(x,y\right)$ is the response at spatial position $(x,y)$, ${c}_{0}$ is the baseline, ${c}_{1}$ is the amplitude, ${c}_{2}$ and ${c}_{4}$ are the RF center in the *x*- and *y*-axis respectively, ${c}_{5}$ and ${c}_{6}$ are the standard deviation (SD) of ${c}_{2}$ and ${c}_{4}$ respectively, and ${c}_{3}$ is the orientation of the elliptical axis. At the beginning, ${c}_{0}$ was calculated as the minimum response and ${c}_{1}$ was calculated as the maximum response. The size of RF was calculated as the mean of twofold SD and further adjusted by calculating dihedral angles. The performance of fitting was evaluated by the adjusted R-square. The averaged RF size was derived from TNs with adjusted R-square larger than zero.

### Statistics

Statistical analysis was performed as depicted in the figure legends by using the GraphPad Prism software. The normality of data was first examined with D’Agostino & Pearson test. For normally distributed data, two-tailed Student’s *t* test, ordinary one-way ANOVA with Tukey’s test for multiple comparisons correction, or two-way ANOVA with Bonferroni’s test for multiple comparisons correction was used. For non-normally distributed data, nonparametric two-tailed Mann–Whitney test or Kruskal–Wallis test with Dunn’s test for multiple comparisons correction was used. The significance of the rhythmicity was estimated by one-tailed Fisher’s *g*-test from the GeneCycle package in R with a dominant period of 24 h^44^. The *P* value < 0.05 was considered to be statistically significant. All results are represented as mean ± s.e.m.

### Reporting summary

Further information on research design is available in the Nature Portfolio Reporting Summary linked to this article.

## Supplementary information

Supplementary Information

Reporting Summary

## Data Availability

The data that support the findings of this study are provided in the Article, Supplementary Information, or Source Data file. Source data are provided with this paper.

## Source data

Source Data

## Code availability

The code for the receptive field analysis, as implemented in MATLAB, is available at https://github.com/soaringdu/Proj-CS.
