# Supplementary material for: Circadian regulation of developmental synaptogenesis via the hypocretinergic system
Source: Nat Commun. 2023 Jun 2;14:3195. doi: 10.1038/s41467-023-38973-w (PMC10238477; doi:10.1038/s41467-023-38973-w)
Supplement: Supplementary file 1 — Supplementary Information [file 41467_2023_38973_MOESM1_ESM.pdf]

Supplementary information

Circadian regulation of developmental synaptogenesis via the hypocretinergic neural system *in vivo*

Du et al.

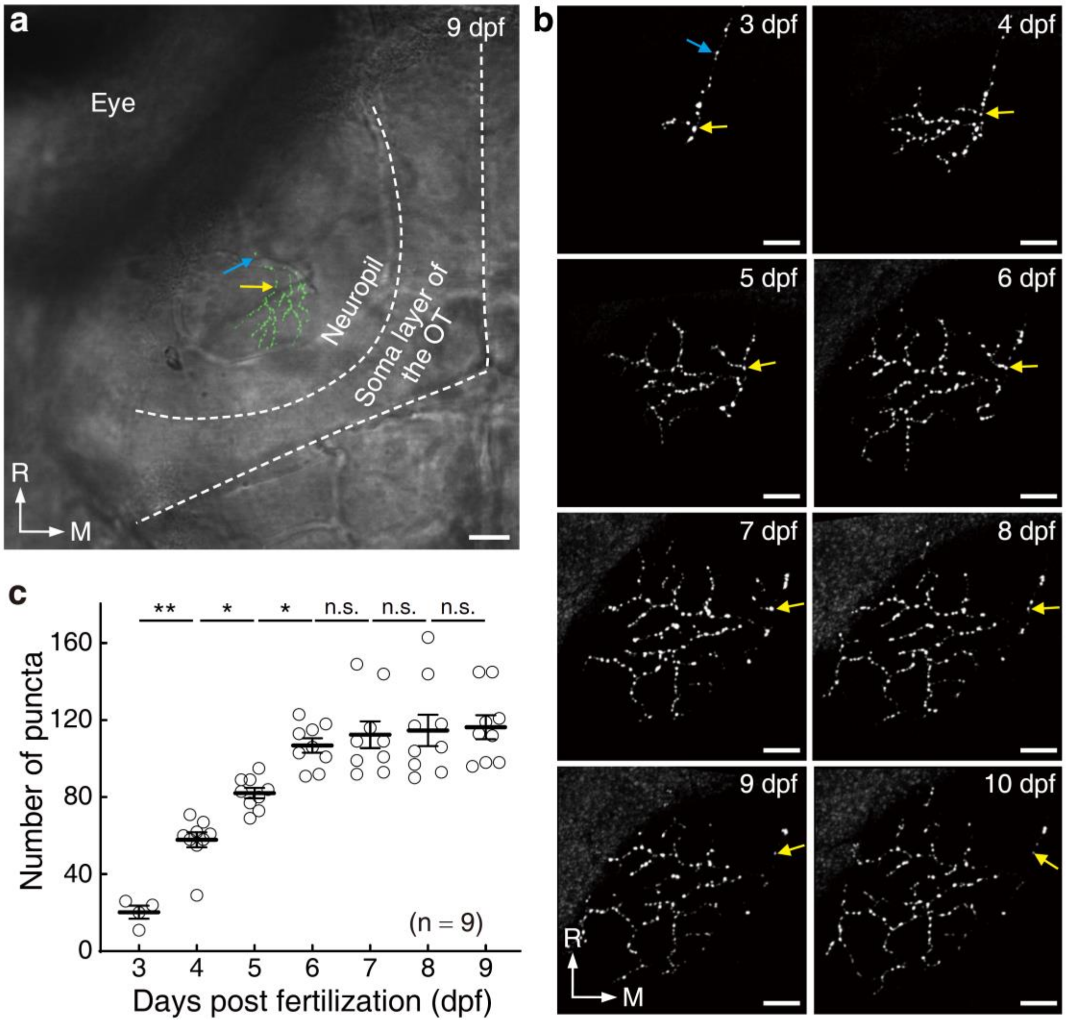

Supplementary Figure 1. Daily change in the number of presynaptic puncta on single-RGC axon arbors *in vivo*. a Dorsal view of the topographic position of a typical RGC axon arbor expressing Sypb-EGFP (in green) in the left tectum of a 9-dpf *PGUSG*. The image was superimposed by one bright-field optical section and a projected two-photon fluorescence image stack. Dotted lines, the boundary between the neuropil and soma layer of the optic tectum (OT) and the borders of the left OT. **b** *In vivo* daily time-lapse two-photon images of the same RGC axon arbor expressing Sypb-EGFP from 3 to 10 dpf. **c** Changes in the total number of puncta on individual RGC axon arbors during 3 to 9 dpf. Among the total of 9 RGCs from 9 larvae examined, 4 RGCs were imaged consecutively from 3 to 9 dpf, and 5 RGCs from 4 to 9 dpf. n.s., not significant; *P = 0.04, **P = 0.007 (one-way ANOVA with Tukey's multiple comparisons test). Error bars denote s.e.m. Scale bars, 20 μm (**a**) and 10 μm (**b**). M, medial; R, rostral. Blue arrow, the primary axon; yellow arrow, the punctum at the first branch point of the RGC axon arbor. Source data are provided as a Source Data file.

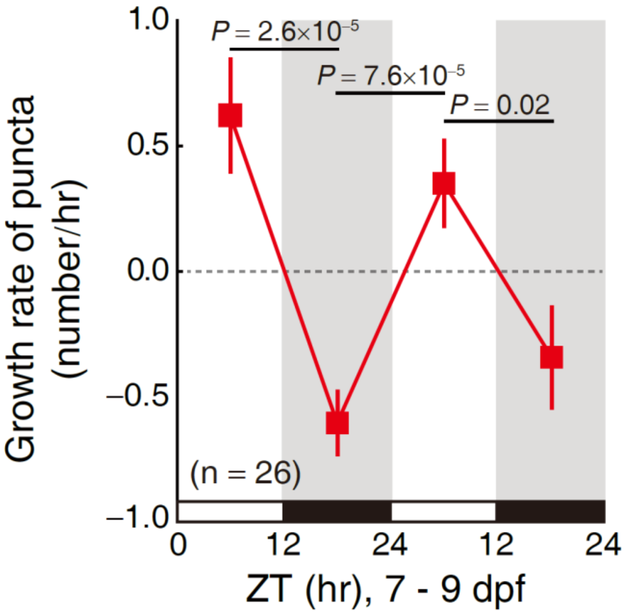

Supplementary Figure 2. Diurnal oscillation of the growth rate of RGC axon puncta during 7 - 9 dpf. Summary of the growth rate of Sypb-EGFP puncta over each 12-hr period across LD cycles during 7 - 9 dpf. Note the change of oscillating level from above zero (see Fig. 1b, red) to near zero from early (4 - 6 dpf) to late (7 - 9 dpf) developmental stages. Among the total of 26 RGCs from 26 larvae examined, 22 RGCs were imaged consecutively from 7 to 9 dpf, 1 RGC from 7 to 8.5 dpf, 2 RGCs from 7 to 8 dpf, and 1 RGC from 7 to 7.5 dpf. ZT, zeitgeber time. Two tailed unpaired Student's *t*-test. Error bars denote s.e.m.. Source data are provided as a Source Data file.

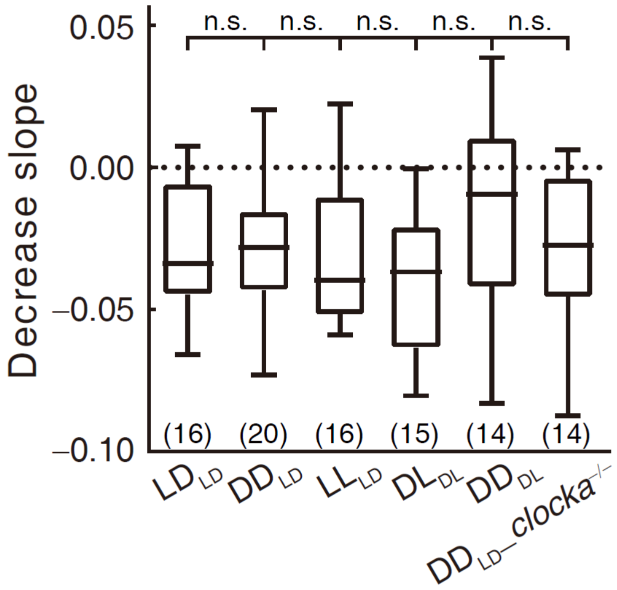

Supplementary Figure 3. Decrease trend of punctum growth rate under different rearing conditions. Boxplots show the decrease slope of the linear fitting line of the dataset (used for analysis in Fig. 1b-g) obtained from larvae reared under different light conditions. The subscript in the *x*-axis labeling represents the light condition used for entrainment during 0 - 4 dpf. n.s., not significant; $P = 0.45$ (Kruskal-Wallis test with Dunn's multiple comparisons test). Centre, median; bounds of box, first and third quartiles; whiskers, minimum and maximum values. LD, light-dark; DD, constant darkness; LL, constant light; DL, dark-light. Source data are provided as a Source Data file.

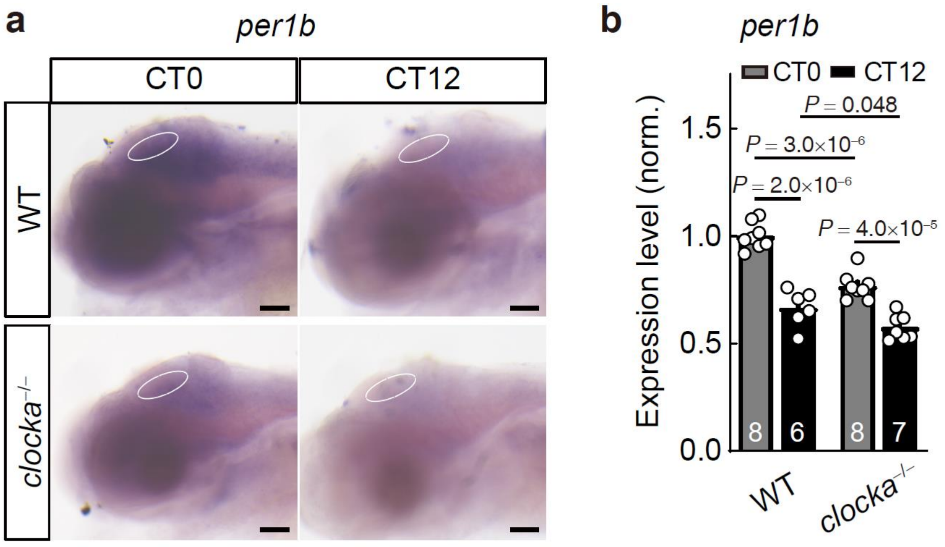

Supplementary Figure 4. The altered expression of clock gene *per1b* in *clocka*$^{-/-}$. a Whole mount *in situ* hybridization showing altered expression of the clock gene *per1b* in the optic tectum of *clocka* mutant under DD conditions after 4-day LD entrainment at the peak (CT0) and trough (CT12) circadian time on 5 - 6 dpf. Scale bars, 50 µm. **b** Quantification of the *per1b* expression in the optic tectum of WT and *clocka*$^{-/-}$. For each individual, the mean signal intensity in the circled tectal area (white circle in **a**) was normalized to the mean value of the WT group at CT0. Nonparametric two-tailed multi *t*-test with Holm-Sidak method for multiple comparisons correction. The numbers on the bars indicate the numbers of larvae examined. Error bars denote s.e.m.. Source data are provided as a Source Data file.

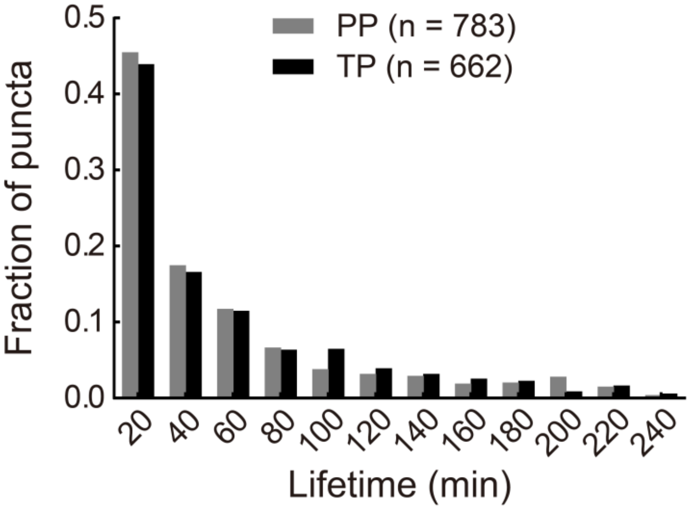

Supplementary Figure 5. Punctum lifespan distribution. Lifetime histogram for a total of 783 and 662 Sypb-EGFP puncta (from 9 Sypb-EGFP-expressing RGCs imaged at 10-minute intervals) observed to form during 4-h time-lapse imaging of 4-5 dpf larvae at day (PP, peak phase) and night (TP, trough phase), respectively. Source data are provided as a Source Data file.

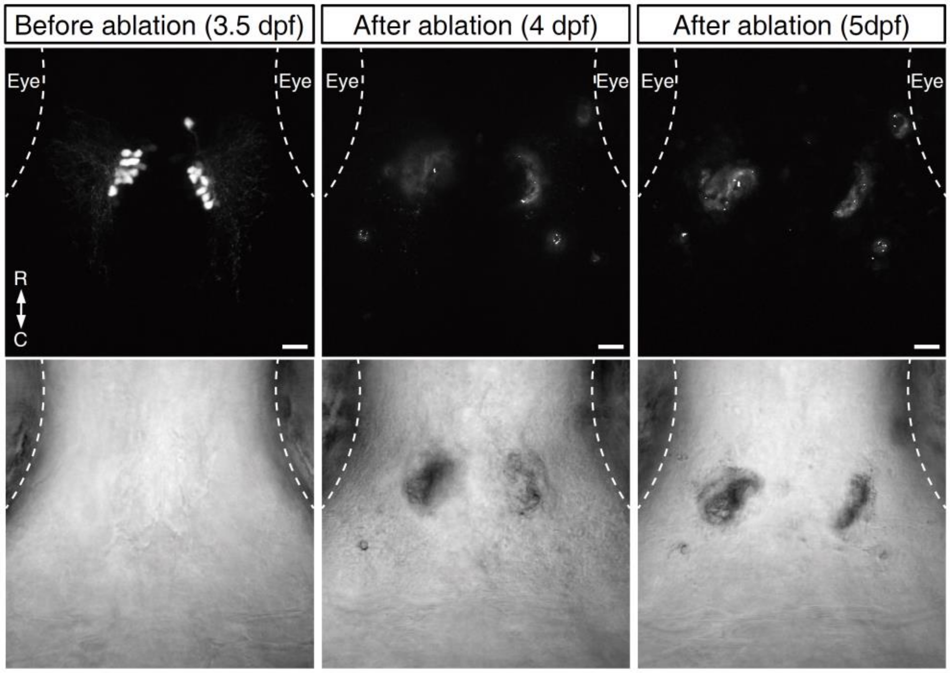

Supplementary Figure 6. Efficiency of two-photon laser-based ablation of bilateral HCRT neurons. Confocal fluorescence (top) and bright-field (bottom) images of HCRT neuron clusters from a single *Tg(-2.0Tru.Hcrt:EGFP)zf11* larva before ablation at 3.5 dpf, and after ablation at 4 and 5 dpf. The ablation effect can be maintained through 4 - 5 dpf during which the synapse growth was examined (see Fig. 3f). C, caudal; R, rostral. Scale bars, 20 μm.

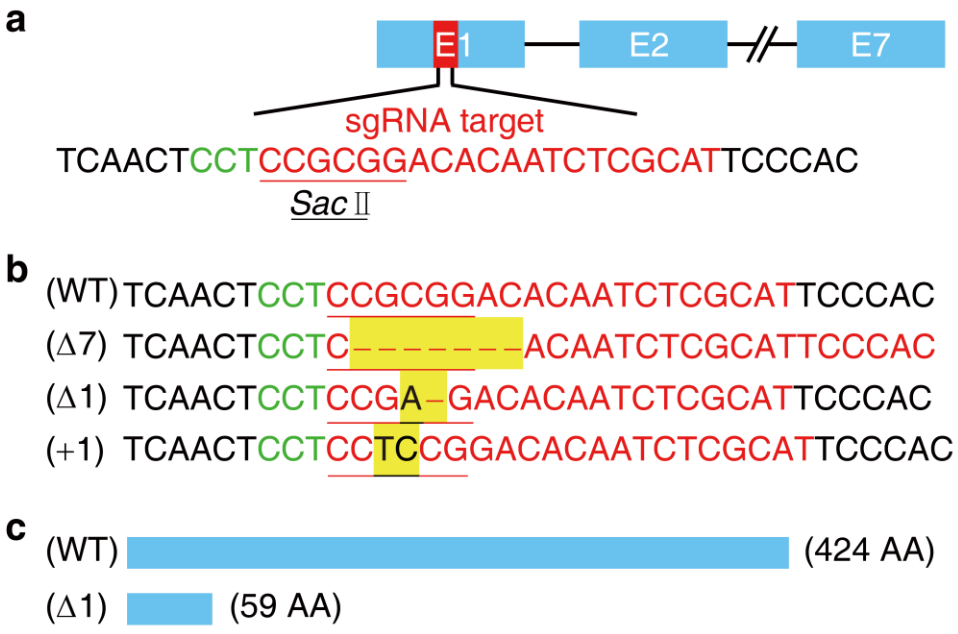

Supplementary Figure 7. *Hcrtr2* gene knockout using the CRISPR/Cas9 system. a Schematic of the sgRNA target site for generating *hcrtr2* knockout at the first exon (E1) of the *hcrtr2* gene. The sgRNA target sequence and the PAM sequence are shown in red and green, respectively. The restriction-enzyme digestion site for genotyping is highlighted as underlined red text. **b** Targeted indel mutations (highlighted in yellow with deletions shown as red dashed lines) induced by sgRNA:Cas9 ribonucleoprotein complex at the *hcrtr2* gene. Three types of mutations were identified. **c** Predicted truncated protein derived from the Δ1 indel mutation used in this study.

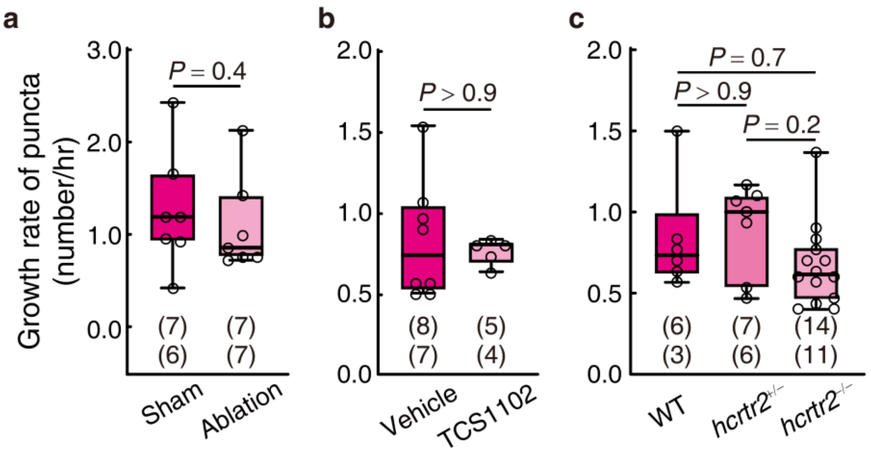

Supplementary Figure 8. Loss of function of the hypocretinergic neural system does not significantly affect the growth rate of presynaptic puncta on RGC axon arbors. a-c Mean growth rate of Sypb-EGFP puncta on single-RGC axon arbors over 30 hrs during which the rhythm of synaptogenic rate was examined (see Fig. 3f-h) with bilateral HCRT neuron ablation (**a**), treatment of the HcrtR antagonist TCS1102 (**b**), or *hcrtr2* mutation (**c**). Two-tailed unpaired Student's *t*-test in (**a**, **b**), Kruskal-Wallis test with Dunn's test for multiple comparisons correction in (**c**). The numbers in the upper and lower brackets indicate the numbers of RGCs and larvae examined, respectively. Centre, median; bounds of box, first and third quartiles; whiskers, minimum and maximum values. Source data are provided as a Source Data file.

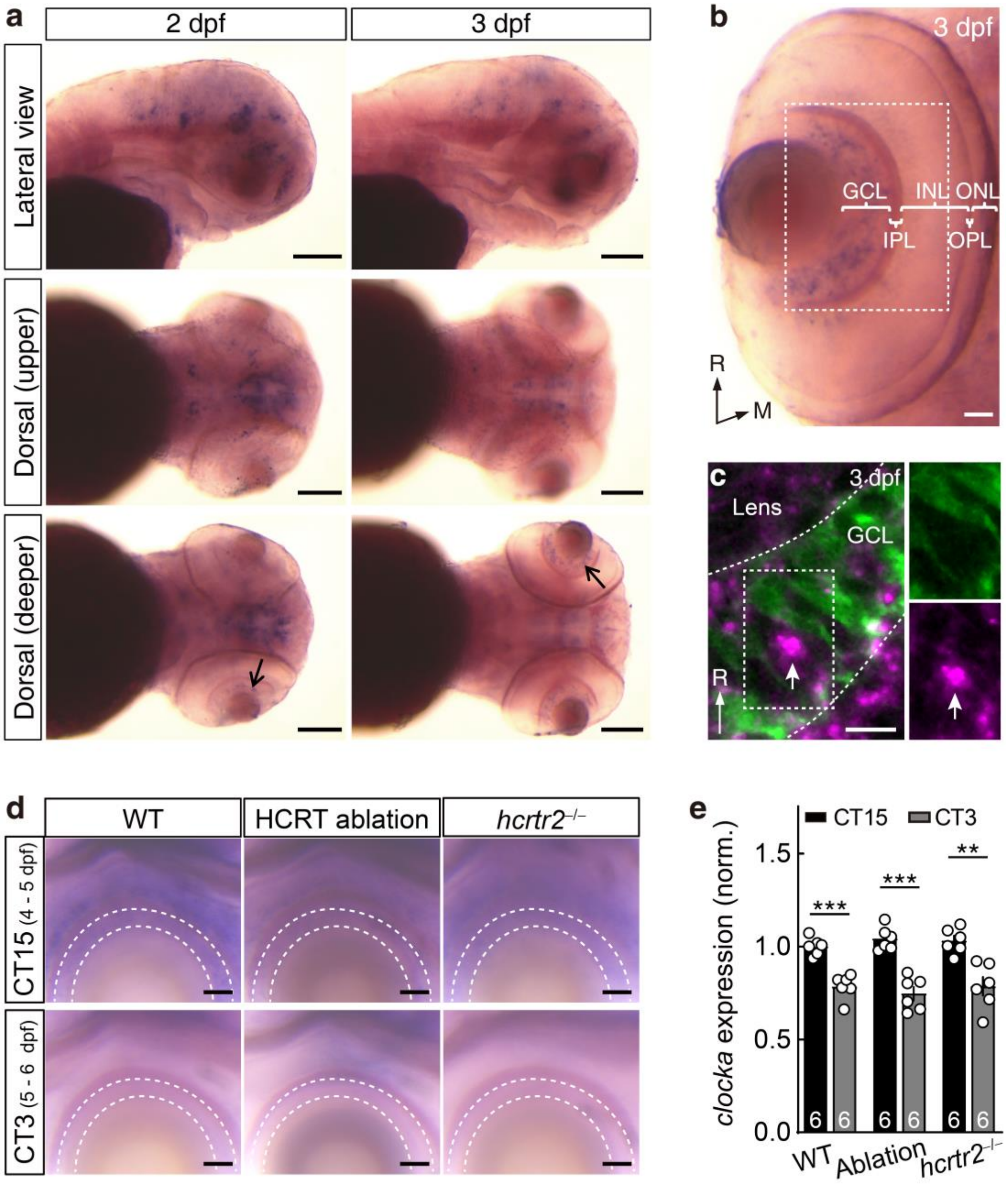

Supplementary Figure 9. The *hcrtr2* expression and the rhythmic *clocka* expression under HCRT signaling disruption in RGCs in larval zebrafish. a Whole-mount *in situ* hybridization (ISH) on 2- and 3-dpf larvae showing *hcrtr2* mRNA expression in several cell clusters in the forebrain, midbrain and hindbrain as previously depicted[23] and also in RGCs in the retina (black arrows). The lateral (top) and two dorsal (middle and bottom) views of the same embryo were shown for each stage. Two independent experiments were repeated. Scale bars, 100 µm. **b** Whole-mount ISH of *hcrtr2* mRNA in a 3-dpf larva showing *hcrtr2* expression in RGCs. GCL, ganglion cell layer; INL, inner nuclear layer; IPL, inner plexiform layer; ONL, outer nuclear layer; OPL, outer plexiform layer. Dotted rectangle denotes the area in retina shown in (**d**). Scale bar, 20 µm. **c** Fluorescence whole-mount ISH of *hcrtr2* mRNA (magenta) and anti-GFP immunostaining

(green) on *Tg(pou4f:GAL4-VP16)ion6d;Tg(4×nrUAS:GFP)c369* fish at 3 dpf showing that *hcrtr2* ISH signals are largely not located on *pou4f3*+ RGCs. Single channel view of the cropped area (dotted rectangle) is on the right. White arrow, a *hcrtr2* ISH cluster. Two independent experiments were repeated. Scale bar, 10 μm. **d** Whole mount ISH showing unaffected rhythmic *clocka* expression under DD conditions after 4-day LD entrainment in the retina of larvae with bilateral HCRT neuron ablation or *hcrtr2* mutation. Scale bar, 20 μm. **e** Quantification of the *clocka* mRNA expression in retina at the two circadian time points shown in (**d**). For each individual, the mean signal intensity in the RGC layer (between dotted lines) at dorsal view was normalized to the mean value of the WT group at CT15. $^{***}P_{\mathrm{WT}} = 7.0\times10^{-5}$, $^{***}P_{\mathrm{Ablation}} = 5.7\times10^{-5}$, $^{**}P = 0.002$ (nonparametric two-tailed multi *t*-test with Holm-Sidak method for multiple comparisons correction). The numbers on the bars indicate the numbers of larvae examined. Error bars denote s.e.m.. M, medial; R, rostral; CT, circadian time. Source data are provided as a Source Data file.

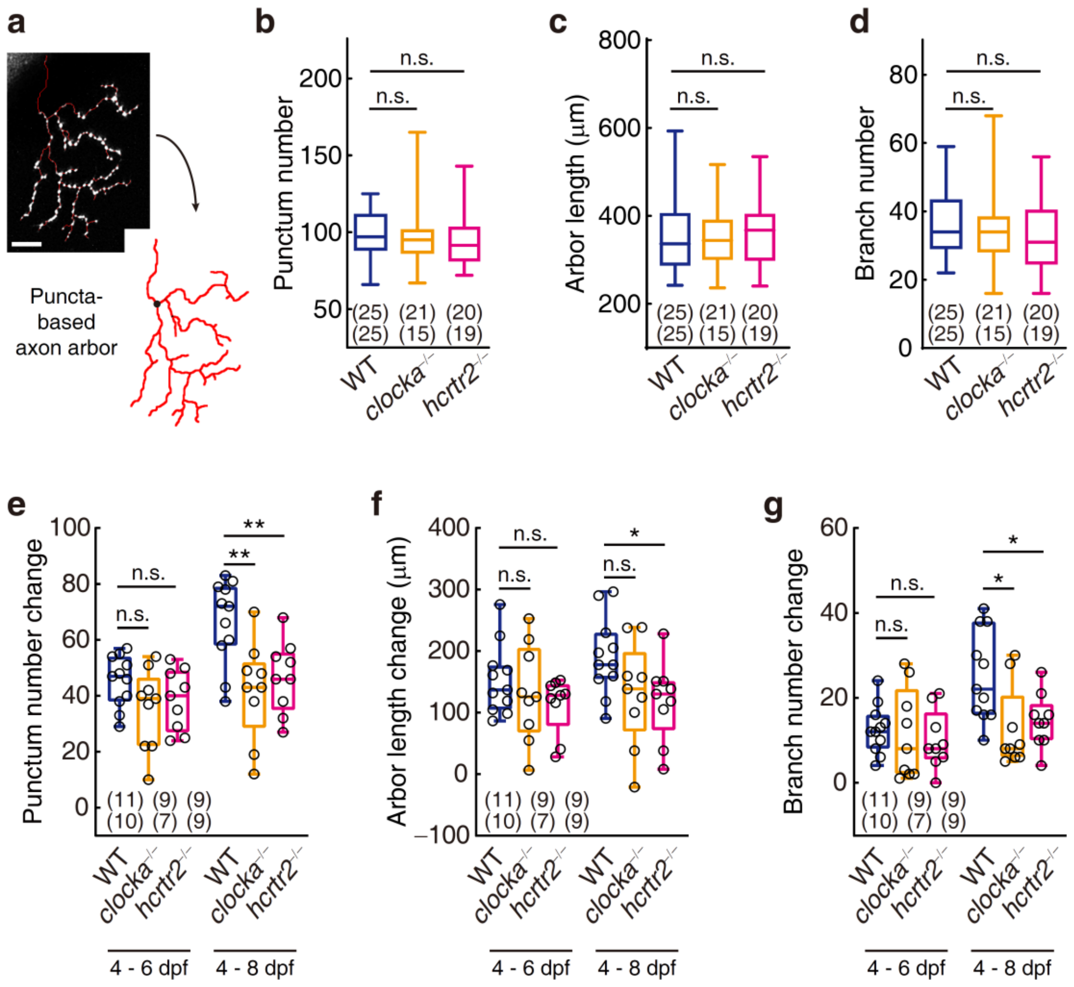

Supplementary Figure 10. Delayed significant change of punctum number, arbor size and arbor complexity in *clocka* and *hcrtr2* mutants. a Upper left, the composite projection view of a typical RGC axon arbor expressing Sypb-EGFP and its reconstructed arbor skeleton (in red) based on punctum signal. Lower right, puncta-based axon arbor tracing. Black dot indicates the first branch point of the axon arbor. Scale bar, 10 μm. **b-d** Boxplots of the number of sypb-EGFP puncta on single-RGC axon arbor (**b**), and of the total length (**c**) and branch number (**d**) of puncta-based single RGC axon arbor at 6 dpf under DD condition after 4-day LD entrainment for WT fish as well as *clocka* and *hcrtr2* mutants. The same datasets were used in (**b-d**) as in Fig. 4b-d. **e-g** Boxplots of the net change of total punctum number (**e**), total arbor length (**f**) and branch number (**g**) from 4 to 6 or from 4 to 8 dpf showing delayed significant effects of circadian disruption on the development of synapse number, axon arbor size and complexity. The same datasets were used in (**e-g**). Numbers in brackets indicate the numbers of RGCs (top) and fish (bottom) examined. n.s., not significant; $**P_{clocka^{-/-}\text{ vs WT}} = 0.004$ and $**P_{hcrtr2^{-/-}\text{ vs WT}} = 0.006$ in (**e**), $*P = 0.02$ in (**f**), $*P_{clocka^{-/-}\text{ vs WT}} = 0.01$ and $*P_{hcrtr2^{-/-}\text{ vs WT}} = 0.02$ in (**g**) (nonparametric two-tailed Mann-Whitney test for **b-d**, nonparametric two-tailed multi *t*-test with Holm-Sidak method for multiple comparisons correction for **e-g**). Boxplots in **b-g**: centre, median; bounds of box, first and third quartiles; whiskers, minimum and maximum values. Source data are provided as a Source Data file.

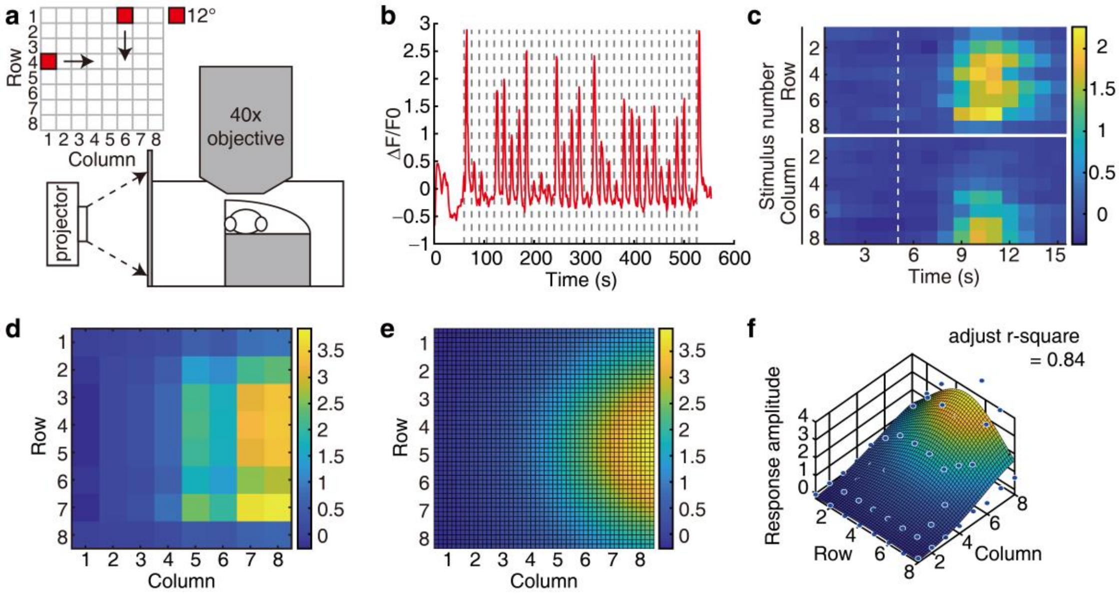

Supplementary Figure 11. Calculation of the receptive field of tectal neurons. a Schematic diagram showing that monocular visual stimuli were projected onto the screen and the contralateral OT was imaged. The visual stimulation field was divided into 8 rows and 8 columns. A red moving square (12 ° in size, 30 °/s in speed) swept along each row and column with a 15-s interval, and each stimulus was randomly repeated twice. The experiment was performed on larvae at 6 dpf under DD conditions after 4-day LD entrainment. **b**, **c** Typical responses of a TN to the moving square stimuli. Both the raw Ca^{2+} activities in response to 32 stimulations (**b**) and the averaged response to 16 stimuli (**c**) were shown. Dashed lines indicate the onset of stimuli. **d-f** Response matrix (**d**) and two-dimensional Gaussian-fitted responses (**e**, **f**) of the neuron shown in (**b**) and (**c**). Source data are provided as a Source Data file.

Supplementary Table 1. Summary of the rhythmicity and phase of synaptogenic rhythm under different experimental conditions. The subscript represents the light condition for entrainment. The rhythmicity test was performed on the detrended data using one-tailed Fisher's *g*-test with a dominant period of 24 hrs (*$P < 0.05$, **$P < 0.01$, ***$P < 0.001$). Significant oscillation (rhythmic) with *P* value less than 0.05 was shown in red. n, number of RGCs examined; N, number of larvae examined; Phase, peak phase.

Conditions	Figure	n	N	*P* value	Phase (hr)	Amplitude
LD_{LD}	Fig. 1c	16	15	0.001**	2.16	0.77
DD_{LD}	Fig. 1d	20	20	0.01*	1.83	0.47
LL_{LD}	Fig. 1d	16	13	0.0006***	1.5	0.47
DL_{DL}	Fig. 1e	15	14	0.006**	9	1.07
DD_{DL}	Fig. 1f	14	13	0.01*	11.83	0.59
DD_{LD}_*clocka*$^{-/-}$	Fig. 1g	14	12	0.3	-	-
DD_{LD}_Sham	Fig. 3d	7	6	0.002**	1.83	0.98
DD_{LD}_HCRT neuron ablation	Fig. 3d	7	7	0.4	-	-
DD_{LD}_Vehicle	Fig. 3e	8	7	0.0008***	2.83	1.08
DD_{LD}_TCS1102	Fig. 3e	5	4	0.08	-	-
DD_{LD}_WT	Fig. 3f	6	3	0.01*	1.83	0.65
DD_{LD}_*hcrtr2*$^{+/-}$	Fig. 3f	7	6	0.06	-	-
DD_{LD}_*hcrtr2*$^{-/-}$	Fig. 3f	14	11	0.2	-	-
