# Supplementary material for: Circadian regulation of developmental synaptogenesis via the hypocretinergic system
Source: Nat Commun. 2023 Jun 2;14:3195. doi: 10.1038/s41467-023-38973-w (PMC10238477; doi:10.1038/s41467-023-38973-w)
Supplement: Supplementary file 2 — Reporting Summary [file 41467_2023_38973_MOESM2_ESM.pdf]

nature portfolio

Corresponding author(s): Jiulin Du, Xufei Du

Last updated by author(s): May 9, 2023

Reporting Summary

Nature Portfolio wishes to improve the reproducibility of the work that we publish. This form provides structure for consistency and transparency in reporting. For further information on Nature Portfolio policies, see our Editorial Policies and the Editorial Policy Checklist.

Statistics

For all statistical analyses, confirm that the following items are present in the figure legend, table legend, main text, or Methods section.

n/a	Confirmed	
☐	☒	The exact sample size (n) for each experimental group/condition, given as a discrete number and unit of measurement
☐	☒	A statement on whether measurements were taken from distinct samples or whether the same sample was measured repeatedly
☐	☒	The statistical test(s) used AND whether they are one- or two-sided<br>*Only common tests should be described solely by name; describe more complex techniques in the Methods section.*
☒	☐	A description of all covariates tested
☐	☒	A description of any assumptions or corrections, such as tests of normality and adjustment for multiple comparisons
☐	☒	A full description of the statistical parameters including central tendency (e.g. means) or other basic estimates (e.g. regression coefficient) AND variation (e.g. standard deviation) or associated estimates of uncertainty (e.g. confidence intervals)
☐	☒	For null hypothesis testing, the test statistic (e.g. F, t, r) with confidence intervals, effect sizes, degrees of freedom and P value noted<br>*Give P values as exact values whenever suitable.*
☒	☐	For Bayesian analysis, information on the choice of priors and Markov chain Monte Carlo settings
☒	☐	For hierarchical and complex designs, identification of the appropriate level for tests and full reporting of outcomes
☐	☒	Estimates of effect sizes (e.g. Cohen's d, Pearson's r), indicating how they were calculated

Our web collection on statistics for biologists contains articles on many of the points above.

Software and code

Policy information about availability of computer code

Data collection	FV10-ASW 4.2 for acquiring confocal images
Data analysis	Image-Pro Plus 6.0.0 for punctum counting and punctum dynamics analysis;<br>Fiji (ImageJ 1.51g) for image time series alignment, in situ signal analysis, axon arbor morphology tracing, and the analysis of RGC axon arborization area and punctum distribution unevenness;<br>L-Measure 5.3 for the quantitative characterization of the length and branch number of RGC axon arbors;<br>AutoQuant X 3.0.5 for image deconvolution in Fig. 1a;<br>GeneCycle package 1.1.2 in R software 3.6.3 for rhythmicity estimation;<br>Custom code written in MATLAB R2018a for the analysis of the receptive fields of tectal neurons are available at https://github.com/soaringdu/CS.;<br>GraphPad Prism 8.3.0 for statistical data analysis.

For manuscripts utilizing custom algorithms or software that are central to the research but not yet described in published literature, software must be made available to editors and reviewers. We strongly encourage code deposition in a community repository (e.g. GitHub). See the Nature Portfolio guidelines for submitting code & software for further information.

Data

Policy information about availability of data

All manuscripts must include a data availability statement. This statement should provide the following information, where applicable:

- Accession codes, unique identifiers, or web links for publicly available datasets
- A description of any restrictions on data availability
- For clinical datasets or third party data, please ensure that the statement adheres to our policy

The data that support the findings of this study are provided in the Article, Supplementary Information, or Source Data file. Source data are provided with this paper.

Research involving human participants, their data, or biological material

Policy information about studies with human participants or human data. See also policy information about sex, gender (identity/presentation), and sexual orientation and race, ethnicity and racism.

Reporting on sex and gender	N/A
Reporting on race, ethnicity, or other socially relevant groupings	N/A
Population characteristics	N/A
Recruitment	N/A
Ethics oversight	N/A

Note that full information on the approval of the study protocol must also be provided in the manuscript.

Field-specific reporting

Please select the one below that is the best fit for your research. If you are not sure, read the appropriate sections before making your selection.

☒ Life sciences ☐ Behavioural & social sciences ☐ Ecological, evolutionary & environmental sciences

For a reference copy of the document with all sections, see nature.com/documents/nr-reporting-summary-flat.pdf

Life sciences study design

All studies must disclose on these points even when the disclosure is negative.

Sample size	No sample size calculation was performed. Sample sizes were chosen based on previous experience with our zebrafish models and are comparable to those in prior studies (Appelbaum et al., Neuron 2010). Sample sizes were provided in Supplementary Table 1 and corresponding figure panels/legend, indicating by n.
Data exclusions	In order to compensate developmental discrepancy of individual RGCs from individual larvae, based on the punctum number distribution at 4 dpf (61.8 ± 13.2, mean ± s.d., n = 72), we selected the axon arbor on which the initial total number of puncta at 4 dpf falls in between 45 to 75 to perform data analysis.
Replication	Each analysis in the current study contained multiple fish from independent experiments (usually 3 - 4) and the numbers of animals used were provided in the Supplementary Table 1 and corresponding figure panels/legends, indicating by N. All attempts at replication were successful.
Randomization	This study did not involve allocation of experimental groups. Data were grouped by mutant phenotype, light condition, circadian time or different treatments.
Blinding	In the study, the investigators were blinded to group allocation during data analysis. Specifically, the investigators were blinded to zeitgeber/circadian time, the phenotype of fish, or treatments.

Reporting for specific materials, systems and methods

We require information from authors about some types of materials, experimental systems and methods used in many studies. Here, indicate whether each material, system or method listed is relevant to your study. If you are not sure if a list item applies to your research, read the appropriate section before selecting a response.

Materials & experimental systems

n/a	Involved in the study	
	x	Antibodies
x		Eukaryotic cell lines
x		Palaeontology and archaeology
	x	Animals and other organisms
x		Clinical data
x		Dual use research of concern
x		Plants

Methods

n/a	Involved in the study	
x		ChIP-seq
x		Flow cytometry
x		MRI-based neuroimaging

Antibodies

Antibodies used	Anti-GFP (Thermo Scientific, A-11122), used at 1:500<br>Goat anti-rabbit Alexa Fluor 488 (Molecular Probes, A11034), used at 1:500 from 2mg/ml stock solution
Validation	Anti-GFP (Thermo Scientific, A-11122) antibody has been validated by Thermo Scientific using immunofluorescent analysis of GFP Tag in H3-GFP construct transfected HEK-293E cells, and also by multiple labs on multiple species including zebrafish (see website).

Animals and other research organisms

Policy information about studies involving animals; ARRIVE guidelines recommended for reporting animal research, and Sex and Gender in Research

Laboratory animals	We used the transgenic fish in nacre background that contain the single or a combination of the following individual driver, reporter, and mutant lines:<br>Tg(pou4f3:GAL4-VP16)ion6d, Tg(UAS:sypb-EGFP)ion7d, clocka-/-, Tg(-2.0Tru.Hcrt:EGFP)zf1120, Mü402321, hcrtr2ion28d, Tg(4×nrUAS:GFP)c369, Tg(elavl3:GCaMP6s)jf4<br>All experimental animals were used at 3 to 9 days post fertilization.
Wild animals	The study did not involve wild animals.
Reporting on sex	The sex of the larval zebrafish used in the study has not yet been differentiated.
Field-collected samples	The study did not involve samples collected from the field.
Ethics oversight	All experimental procedures using live animals were performed in accordance with guidelines approved by the Animal Care and Use Committee of the Center for Excellence in Brain Science and Intelligence Technology, Chinese Academy of Sciences (NA-046-2023).

Note that full information on the approval of the study protocol must also be provided in the manuscript.
